# Serotonin Syndrome: The Role of Pharmacology in Understanding Its Occurrence

**DOI:** 10.7759/cureus.38897

**Published:** 2023-05-11

**Authors:** Leila R Poian, Silvana Chiavegatto

**Affiliations:** 1 Department of Pharmacology, Biomedical Sciences Institute, University of Sao Paulo (ICB-USP), Sao Paulo, BRA; 2 Department of Psychiatry, Institute of Psychiatry, University of Sao Paulo Medical School (FMUSP), Sao Paulo, BRA

**Keywords:** serotonin syndrome diagnosis, selective serotonin reuptake inhibitor (ssri), antidepressants, diagnostic criteria, genetic polymorphisms, toxidromes, atypical neuroleptic malignant syndrome, neuroleptic malignant syndrome (nms), serotonin toxicity, serotonin syndrome (ss)

## Abstract

Serotonin syndrome (SS) is a potentially fatal adverse drug reaction characterized by an exaggerated increase in serotonergic activity in the central and peripheral nervous systems. It presents a constellation of signs and symptoms related to behavioral changes, neuromuscular excitability, and autonomic instability. These symptoms can occur in both mild and severe forms. SS can be triggered by the therapeutic use of a drug that increases serotonin (5-HT) availability in the synaptic cleft or by the co-administration of two or more drugs that provide this increase. With the escalating use of antidepressants by the world's population, this adverse reaction may be more recurrent. However, SS is often overlooked by patients or not diagnosed by doctors. This review aims to improve awareness about SS and provide a pharmacological perspective to explain its occurrence. Evidence shows that other neurotransmitters may also be involved with the pathology of SS. Furthermore, SS and neuroleptic malignant syndrome (NMS) seem to be part of the same pathological spectrum, especially in atypical NMS cases. The emergence of the syndrome's symptoms may be closely related to pharmacokinetic and/or pharmacodynamic polymorphisms that lead to an increase in the 5-HT available to or 5-HT signaling by specific receptors, thus constituting an important area for future investigations.

## Introduction and background

Serotonin syndrome (SS), which also can be referred to as serotonin toxicity or serotonin toxidrome, is an adverse drug reaction characterized by an exaggerated increase in serotonergic activity in the central and peripheral nervous systems (CNS and PNS, respectively) [1,2]. It comprises a constellation of signs and symptoms related to behavioral changes, neuromuscular excitability, and autonomic instability. These symptoms can occur in mild, severe, and potentially fatal forms [1]. This syndrome can occur with the therapeutic use of a drug that increases the availability of serotonin (5-hydroxytryptamine; 5-HT) in the synaptic cleft, usually an antidepressant of the selective 5-HT reuptake inhibitor (SSRI) class, or by the interaction of two or more drugs that provide this 5-HT increase [1,3].

Due to the lack of medical awareness about SS, its true incidence is unknown and difficult to measure [4-6]. In 2017, the "Toxic Exposure Surveillance System" reported 57,254 cases of exposure to SSRIs in the US (of which 24,819 or 43.3% of cases were single exposures), with 5 or 0.009% of these cases culminating in death [7]. According to the World Health Organization [8], the global number of people with depression increased by 18.4% from 2005 to 2015. Since the effectiveness of antidepressant drugs is considered suboptimal, associated with the slow development of new drugs in this class, knowing as much as possible about all aspects of existing drugs becomes paramount [9].

Another current aggravating factor, which further increases the need for attention and consciousness about SS, is the worsening mental health of the population due to the COVID-19 pandemic. In a cohort study, which used information obtained from the US Veterans Health Administration database, three groups of patients were compared: (1) patients who had COVID-19; (2) patients who did not test positive for the disease during the same period; (3) control group with patients who had influenza in the pre-pandemic period. The results suggest that the first group had a greater risk of developing various mental health disorders such as anxiety, depression, opioid and other substance use, sleep, and stress disorders [10]. In Brazil, the cohort study "Brazilian Longitudinal Health Study" (ELSA-Brasil) investigated the psychopathological changes generated by COVID-19 in 2,011 patients (those who were from the beginning of the study in 2008 and who remained until the year 2020). The results suggested that the level of depressive disorder remained stable during the pandemic, but this level was already considered very high, affecting about 30% of the current population [11]. Indeed, there is evidence of increased prescription and consumption of antidepressants, especially SSRIs, in several countries in Europe and North and Latin America [12].

Given the lack of medical awareness about the syndrome associated with the increased use of antidepressants with several drug interactions that can trigger it, this review aimed to improve the current understanding of SS occurrence using a pharmacological mechanistic approach. Specifically, we analyzed the underlying pharmacological mechanisms that contribute to the development of this syndrome and the drug interactions that can lead to it. Through this approach, we hope to provide new insights to identify and prevent this life-threatening condition.

## Review

History of serotonin syndrome

The discovery of the antidepressant properties of drugs like iproniazid and imipramine in the 1950s transformed the treatment of depression and anxiety. Iproniazid, initially developed for tuberculosis, was found to inhibit the enzyme monoamine oxidase (MAO), and this inhibition was believed to be responsible for its antidepressant effects [13]. Similarly, imipramine, which was originally being investigated as an antipsychotic drug, was found to have significant antidepressant properties [14].

Oates and Sjoerdsma, in 1960, were the first to relate specific clinical manifestations to increased 5-HT levels in the CNS. They observed a syndrome characterized by nervousness, diaphoresis, mental dysfunction, ataxia, and hyperreflexia in patients who were taking an MAO inhibitor (MAOI) while on antihypertensive treatment with L-tryptophan. This syndrome was later named the "Indolamine Syndrome" [15]. In 1975, researchers observed abnormal behavior in male rats treated with serotonergic agonists, which they called the "5-HT Behavioral Syndrome" [16]. The term "Serotonin Syndrome" or "5-HT Syndrome" was coined by Gerson and Baldessarini in 1980, in their review of the clinical features and etiology of the syndrome [17].

Serotonin system

**Figure 1:**
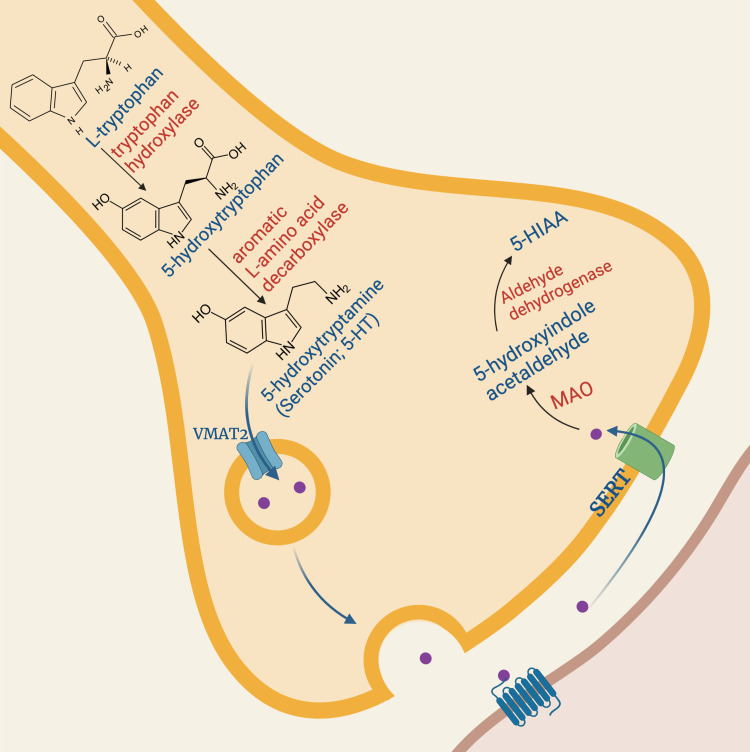
5-HT synthesis and degradation The cell bodies of serotonergic neurons are in the raphe nuclei in the brainstem, and their axons propagate and extend widely throughout the brain and spinal cord. 5-HT biosynthesis occurs in these cell bodies and begins with the passage of the essential amino acid L-tryptophan across the blood-brain barrier via a non-tryptophan-selective amino acid transporter [25]. The first step and the rate-limiting factor in 5-HT synthesis is the hydroxylation of L-tryptophan to 5-hydroxytryptophan (5-HTP) by the enzyme tryptophan hydroxylase (found only in serotonergic neurons). Then, decarboxylation occurs by the enzyme L-aromatic amino acid decarboxylase, generating the final compound 5-hydroxytryptamine [25]. After synthesis, 5-HT is rapidly transported to storage vesicles via a vesicular monoamine transporter (VMAT) isoform 2 [25]. There are two isoforms of VMAT: 1 and 2. VMAT1 is mostly in neuroendocrine cells, and VMAT2 is in monoaminergic neurons of the CNS and PNS [26]. Free 5-HT in the synaptic cleft is transported back to the presynaptic neuron via the 5-HT reuptake transporter (SERT; 5-HTT). The MAO enzyme degrades 5-HT. There are two isoforms of MAO (A and B); MAO-A more selectively degrades 5-HT, while MAO-B nonspecifically degrades catecholamines [27].

5-HT is a monoamine present in most organic systems, but its best-known action is as a neurotransmitter molecule in the CNS [18]. It is responsible for regulating neurobehavioral processes such as attention, sleep-wake, appetite [19], affective and sexual behaviors [20], thermoregulation [21], aggressive behavior [22], nociception, emesis, and motor tone [18]. In the periphery, 5-HT is produced by enterochromaffin cells distributed throughout the gastrointestinal (GI) tract [23,24]. It is estimated that more than 90% of circulating 5-HT is produced by these cells [24]. Figure 1 illustrates the process of serotonin synthesis in detail.

Currently, 14 5-HT receptors have already been cloned and are classified into seven families of receptors (5-HT1 to 5-HT7) with different subtypes [28]. Table 1 describes the distribution and effects of each 5-HT receptor.

**Table 1:** 5-HT receptors and their distribution and effects

Receptor Family	Receptor Subtypes	Signaling Mechanisms	Distribution	Effects
5-HT1	5-HT1A 5-HT1B 5-HT1D 5-HT1e 5-HT1F	G-protein coupled - Inhibit adenylate cyclase	Prefrontal cortex [28], raphe nuclei (autoreceptors), and the terminal regions as postsynaptic sites (mainly in cortico-limbic areas) [29]	5-HT1A: anxious behavior [18] 5-HT1B/D: anti-migraine properties and an antiplatelet effect [18,30]
5-HT2	5-HT2A 5-HT2B 5-HT2C	G-protein coupled - Activates phospholipase C	5-HT2A: frontal cortex, nucleus accumbens, midbrain nuclei, hippocampus [31], smooth muscle, and platelets [18]	5-HT2A: induces hallucinations (in high 5-HT concentration) in the prefrontal cortex [28]; the antagonism is an established treatment for schizophrenia [31,32]
5-HT3	5-HT3A (single functional isoform)	Non-selective cation channel	Dorsal vagal complex, hippocampus, frontal cortex, cingulated cortex, ventral tegmental area, and immune cells [28]	5-HT3A antagonism has an antiemetic effect [23] and may decrease the behavioral consequences of withdrawal syndromes caused by the treatment of drugs of abuse and for several psychiatric disorders (e.g., psychoses, anxiety, and cognitive dysfunction) [28,33]
5-HT4	5-HT4A 5-HT4B 5-HT4C 5-HT4D	G-protein coupled - Activates adenylate cyclase	5-HT4A-B-C are found in atrium, brain and intestine; 5-HT4A in bladder; 5-HT4B in the kidneys; and 5-HT4D in intestinal cells [34]	Modulate the motility and secretory response of the GI tract [23,28]
5-HT5	5-HT5A 5-HT5B	G-protein coupled - Inhibit adenylate cyclase	The prefrontal cortex, hippocampus, amygdala, cerebellum, striatum, and substantia nigra [28]	5-HT5A receptors have a distribution that could theoretically modulate emotion regulation, cognition, and nociception; however, due to the lack of selective substances for these receptors, none of these functions could be tested [28]
5-HT6		G-protein coupled - Activates adenylate cyclase	Hypothalamus, hippocampus, and cerebral cortex [28]	Some antidepressants and antipsychotics have an affinity for these receptors. Still, they also show selectivity for other targets, making it difficult to define the functions of this family of receptors [28]
5-HT7		G-protein coupled - Activates adenylate cyclase	Hypothalamus, thalamus, and hippocampus. Smooth muscle cells, blood vessels, and gastrointestinal tract [28]	Seems to have a pro-depressive effect. Some preclinical studies in animal models using an unspecific antagonist suggest a significant role in depression [35]

Pathophysiology of serotonin syndrome

**Figure 2:**
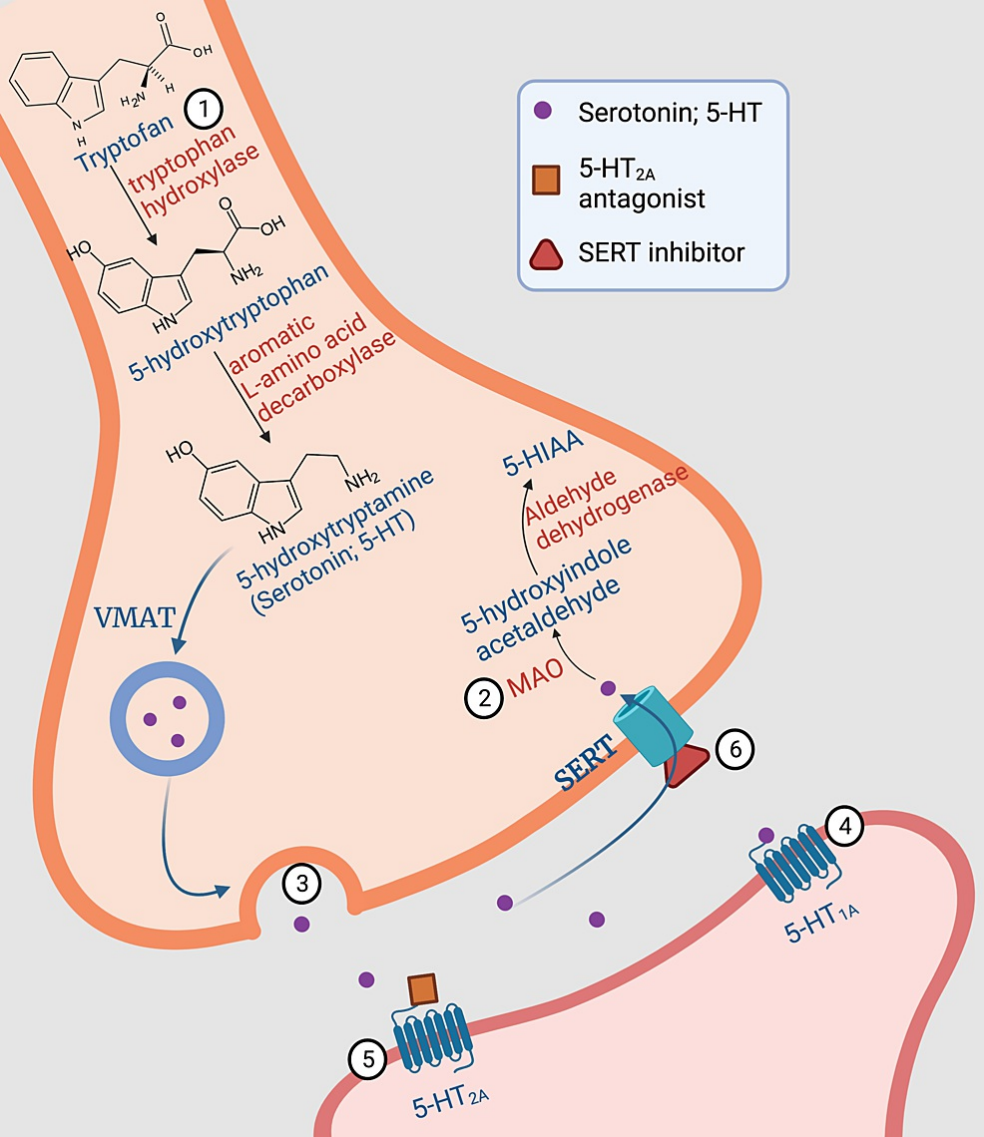
Possible mechanisms for the occurrence of serotonin syndrome 1 - Increased levels of the 5-HT precursor, L-tryptophan; 2- Inhibition of monoamine oxidase; 3- Increased release of 5-HT by drugs such as amphetamine, cocaine, MDMA and levodopa; 4- Activation of the 5-HT1A receptor directly or indirectly; 5- 5-HT2A receptor antagonism; 6 – Inhibition of serotonin reuptake by SERT blockade. Adapted from [36], [37] and [38].

Six possible mechanisms for the occurrence of SS are listed below and illustrated in Figure 2.

Increased Availability of the Essential Amino Acid L-Tryptophan

Theoretically, an increased supply can lead to an increment in endogenous 5-HT. The study that investigated this risk factor used patients who also used MAOIs, which are drugs that make up the second mechanism for the occurrence of SS [15]. Thus, an interaction between these factors leading to SS cannot be excluded.

Inhibition of MAO

By inhibiting MAO, 5-HT is once again stored in the vesicles of the presynaptic neuron and subsequently released into the synaptic cleft to perform its function, which allows for a longer duration of action [36].

Promotion of 5-HT Release

Some illicit substances, such as amphetamine, 3,4-methylenedioxymethamphetamine (MDMA), and cocaine can promote the release of 5-HT into the synaptic cleft and thus lead to syndrome onset [39]. It has also been described that levodopa can be decarboxylated into dopamine (DA) in serotonergic neurons. This newly synthesized DA competes with 5-HT for VMAT, after which it is released into the synaptic cleft along with 5-HT. Furthermore, this DA can hinder the entry or exit of 5-HT through the SERT [40,41].

Postsynaptic Receptors

The fourth and fifth mechanisms depend on the interaction of 5-HT and/or agonist or antagonist drugs with their postsynaptic receptors. They can also occur through increased availability of 5-HT in the cleft (also drug-mediated) [36,38].

SERT Blockade

Another way to prolong the action of 5-HT in the synaptic cleft is to inhibit its reuptake into the presynaptic neuron by blocking SERT [36].

5-HT receptors involved in serotonin syndrome

The symptoms that characterize SS can be mainly related to the agonism and/or antagonism of two subtypes of receptors: 5-HT1A and 5-HT2A [36,39]. There is no single receptor responsible. The 5-HT1A receptors seem to contribute to symptoms such as myoclonus, hyperreflexia, vasomotor tone alterations, anxiety, increased respiratory rate, and hyperactivity [1,39]. This receptor has been associated with several behaviors consistent with SS in rats and mice, such as Straub's tail, walking backward, stepping with forepaws, head shaking, tremors, and hindlimb abduction [42]. Accordingly, these behaviors are more apparent in mice overexpressing 5-HT1A receptors [43].

Another receptor that appears closely related to the syndrome is the 5-HT2A receptor. The appearance of the most severe SS symptoms may be related to the agonism of these receptors, as its stimulation can cause platelet aggregation, bronchoconstriction, vasoconstriction (and consequent hypertension), and the behavioral and cognitive changes characteristic of the syndrome [1,37,44]. This receptor has also been linked to SS-associated hyperthermia in animal models. Administration of the antipsychotic olanzapine (5-HT2A receptor antagonist) in an SS animal model (Wistar rats treated with fluoxetine 10 mg/kg + tranylcypromine 3.5 mg/kg) prevented the onset of hyperthermia, which supports the hypothesis of the involvement of these receptors on body temperature [45].

On the other hand, the antagonism of 5-HT2A receptors also seems to be associated with SS onset [46]. A 2018 study explored this topic with a bioinformatics approach, using the FDA Adverse Reactions Reporting System (FAERS) and pharmacokinetic data from the literature. They reported that when associated with SSRIs, second-generation antipsychotics exert an effect that may reflect 5-HT2A receptors antagonism with simultaneous activation of 5-HT1A receptors, which might be an SS onset mechanism for these two classes of drugs [38].

The two receptors, 5-HT1A and 5-HT2A, are extensively co-expressed in the prefrontal cortex (PFC) [28,29,47]. When SERT is inhibited, there is an increase in 5-HT in this brain area. Increased 5-HT will consequently elicit an excitatory effect through 5-HT2A receptor activation and an inhibitory effect caused by 5-HT1A receptor activation in pyramidal neurons, which project to other cortical areas and subcortical regions such as the limbic system [29,47]. By adding 5-HT2A receptor antagonists in this scenario, there will be an increase in neurotransmission mediated by postsynaptic 5-HT1A receptors, therefore modifying the control made by the PFC in the corticolimbic networks [29,47,48].

**Figure 3:**
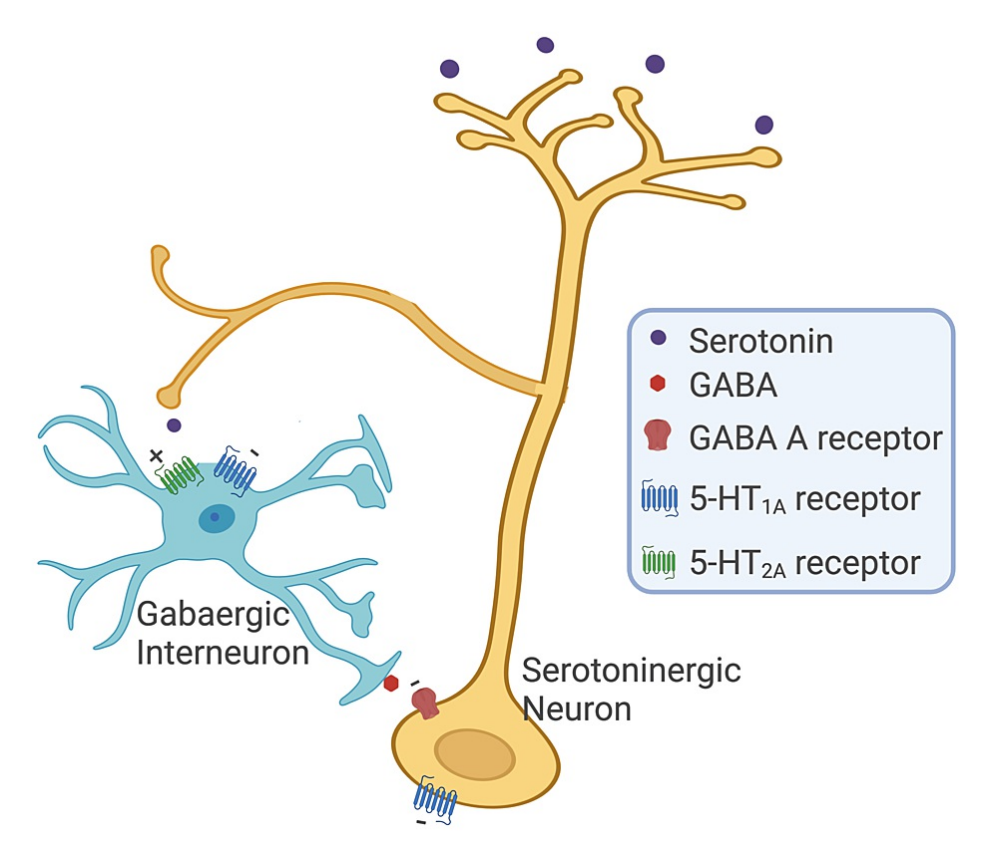
Schematic representation of the negative feedback in the GABA and 5-HT circuit Adapted from [49]. Created with Biorender.com

Moreover, the antagonism of 5-HT2A receptors is suggested to be implicated in the increase of serotonergic neurotransmission by inhibiting GABAergic interneurons in the dorsal raphe nucleus [49]. As shown in Figure 3, the GABAergic interneurons located in the dorsal nucleus of the raphe and periaqueductal gray matter are stimulated by 5-HT through 5-HT2A/2C receptors and inhibited by 5-HT1A receptors also present there. This activation promotes the release of GABA, which inhibits subsequent serotonergic neuron stimulation, blocking 5-HT release. When administered with a 5-HT2A/2C receptor antagonist, 5-HT will bind only to 5-HT1A receptors. This binding promotes the inhibition of GABAergic interneurons, which will not release GABA, and, consequently, will promote the release of 5-HT by serotonergic neurons [49].

A SS animal model, using substances with a strong affinity for 5-HT1A receptors like 8-OH-DPAT and gepirone, was developed utilizing male Sprague-Dawley rats (termed "behavioral syndrome" in rodents) [50]. After inducing the behavioral syndrome, these animals were treated with nonspecific 5-HT2/5-HT1C receptor antagonists (ritanserin, ICI 170,809, and ketanserin), and it was observed that the blockade caused by these agents increased the behavioral syndrome [50]. The authors suggest that 5-HT exerts an inhibitory effect on 5-HT1A receptor functions through 5-HT2 receptors or possibly through 5-HT1C receptors. Thus, blocking these receptors may release the 5-HT1A receptors from the inhibitory influence [50].

The activation of 5-HT3 receptors is related to SS symptoms such as diarrhea, nausea, and abdominal pain. Thus, 5-HT3 receptor antagonists have clinical relevance in relieving nausea and emesis, mainly those resulting from cancer treatment, as mentioned above. Some studies have reported SS cases associated with these antagonists, but the exact mechanism has yet to be elucidated [51].

Occurrences of serotonin syndrome

It is known that SS can occur by an overdose of a drug that increases 5-HT availability or because of a drug interaction between two or more drugs at therapeutic doses (more common in clinical practice). Generally, in SS cases caused by a single drug, the symptoms are moderate, and there is a better prognosis. In contrast, when SS is triggered by drug interactions, mainly by MAOIs and SSRIs, the symptoms are more severe and serious and can lead to death [44,52].

Other neurotransmitters may also be involved in developing the severe form of SS [37]. For example, 5-HT has been shown to cause direct stimulation of neurotransmitter release (e.g., GABA, glutamate, and DA) by the dorsal raphe nuclei [25,37]. It is also suggested to influence noradrenaline (NA) release by the hypothalamus in animals [53]. Indeed, previous studies showed that SS causes a hyperadrenergic state that culminates in physiological stress [54,55]. In this sense, some case reports consider SS an inducer of Takotsubo syndrome [54-56], characterized by transient left ventricular dysfunction caused by physical and/or emotional stress, without the individual having previous heart disease [56]. Additionally, NA is known for its effect on blood pressure, in addition to causing tachycardia, tachypnea, tremor, and agitation [57].

Drugs associated with serotonin syndrome

According to the data found in the literature, the drugs capable of developing SS alone are all SSRIs and nefazodone [2,6,57]. On the other hand, 5-HT3 and 5-HT2A receptor antagonists and opioids can cause SS when used concomitantly with any antidepressant. Drugs, such as lithium and methylphenidate, do not have confirmed affinity or activity in any of the serotonergic receptors, yet several case reports associate their use with SS [58,59]. These drugs appear to increase 5-HT release and postsynaptic 5-HT1A receptor sensitivity [60,61]. Table 2 describes the drugs associated with SS according to their mechanisms. 

**Table 2:** Drugs associated with SS according to their mechanisms *Lithium - as explained earlier in the text, it does not seem to have proven action on serotonergic receptors. Adapted from  [2,37,38,57].

Mechanism	Drugs associated with SS
Increased 5-HT synthesis	Food supplement: tryptophan
Inhibition of 5-HT metabolism	MAOIs: selegiline, rasagiline, tranylcypromine, linezolid (antibiotic), methylene blue
Increased 5-HT release	Amphetamine and derivatives: amphetamine, methylphenidate, cocaine, MDMA. Lithium*
5-HT_1_ receptor agonism	Triptans: sumatriptan, naratriptan, rizatriptan, Opioids: fentanyl, meperidine, sufentanil. Antidepressants and mood stabilizers: mirtazapine, trazodone, lithium*
5-HT_2A_ receptor antagonism	Second-generation antipsychotics: quetiapine, risperidone, olanzapine, clozapine, aripiprazole, and ziprasidone Antidepressant: nefazodone
Inhibition of 5-HT reuptake (SERT) from the synaptic cleft	SSRI: citalopram, escitalopram, fluoxetine, fluvoxamine, paroxetine, sertraline 5-HT and NA reuptake inhibitors (SNRIs): venlafaxine, duloxetine, desvenlafaxine Tricyclic antidepressants (TCA): amitriptyline, clomipramine, desipramine, doxepin, imipramine, nortriptyline Multimodal antidepressant: vortioxetine. Opioids: meperidine, methadone, tramadol, codeine, oxycodone, morphine 5-HT3 receptor antagonists: ondansetron and granisetron. Antidepressants and other classes: trazodone, nefazodone, and cyclobenzaprine.

Drugs associated with SS interact with several receptors which go beyond their desired initial interaction. Culbertson et al. (2018) demonstrated additional targets than those described in the technical information leaflets using a bioinformatics approach. They show how these interactions are diverse and with different levels of affinities. For example, naratriptan is a high affinity 5-HT1 receptor subfamily agonist, with a moderate affinity towards 5-HT2A, 5-HT2C, 5-HT3, and 5-HT7 receptors, with no evidence of activity on these receptors being as agonists or antagonists. In addition, this drug has also been shown to have a moderate affinity for SERT, managing to exert a critical inhibitory activity [57]. This study also showed that the additional antagonistic activity of some drugs on muscarinic acetylcholine receptors might result in more SS cases than with drugs inhibiting NET and SERT alone [57].

Clinical conditions associated with serotonin syndrome

The risk of developing SS appears to be associated with kidney diseases that may predispose the patient due to impaired drug clearance, which circulates longer in the individual's body [37]. Some studies have shown that there is an increase in circulating 5-HT in patients with cardiovascular diseases such as hypertension [62,63], thrombosis [44,64], myocardial infarction, and coronary atherosclerosis [64,65], and in patients who use tobacco [66].

Hepatic metabolism plays an essential role in developing several drug interactions. Cytochrome P450 (CYP450) is a family of oxidative isoenzymes found in the microsomes of various tissues (e.g., liver and intestines) responsible for metabolizing several drugs. The drugs involved in SS are mainly metabolized by the isoenzymes CYP2C19, CYP2D6, and CYP3A4 [67]. Some factors, such as age, sex, and some diseases, contribute to natural variations in the metabolism performed by these isoenzymes. Particular attention should be paid to elderly patients, who should have SS as a differential diagnosis for cases of mental status changes [67].

Interestingly, changes in the pharmacokinetic parameters of SSRIs leading to adverse reactions have been associated with polymorphisms in CYP2D6 and CYP2C19 [9,68,69], mainly in slow metabolizers and intermediate metabolizers, as shown by several case reports [70-73]. A study with 100 patients treated with only venlafaxine (orally), within the recommended doses for major depressive disorder, identified 25 with polymorphisms in CYP2D6 (at least one defective allele) responsible for most of the metabolism of venlafaxine. Four patients were classified as poor metabolizers, with significantly slower venlafaxine metabolization than normal or fast metabolizers. These individuals also had more adverse reactions than the others, showing that poor metabolizers have a greater risk of having adverse reactions to venlafaxine at normal doses [74].

In a recent systematic review and meta-analysis carried out with data from 8,379 individuals genotyped for CYP2D6 and CYP2C19, using any of the 14 drugs analyzed (antipsychotics and antidepressants), it was possible to observe that patients with polymorphisms in CYP2D6 had more adverse effects when administered aripiprazole, haloperidol, risperidone, nortriptyline, paroxetine, quetiapine, amitriptyline, mirtazapine, or fluvoxamine. Additionally, patients with CYP2C19 polymorphisms exhibited more effects from escitalopram, sertraline, clozapine, fluoxetine, and venlafaxine [9]. These polymorphisms could explain why many patients can take several serotonergic drugs without experiencing adverse reactions.

Another interesting variable is 5-HT2A receptor polymorphisms found in some individuals [75]. Genetic variations in this receptor can alter the membrane surface expression, signal transduction, and ligand affinity [76]. One example is the T102C polymorphism located in the first exon of the 5HTR2A gene. The thymine (T) for cytosine (C) substitution does not significantly alter the amino acid sequence (i.e., silent polymorphism); however, it is positioned close to the promoter region and could affect gene and/or protein expression [77]. Indeed, a study with human post-mortem tissue observed that the presence of the C allele reduced the production of 5-HT2A receptors by 20% in the temporal cortex [78]. Some authors suggest a correlation between homozygous individuals for the C102 allele of the 5-HTR2A gene and a greater risk of developing adverse reactions related to 5-HT [79,80].

However, in a study carried out with 95 patients who overdosed on various antidepressant classes, a correlation between polymorphisms in the C102 allele of the 5-HTR2A gene with a higher risk of developing SS was not observed [75]. It is important to emphasize that only Hunter's criteria were used to diagnose SS in those patients, limiting the diagnosis of the syndrome to only 14 participants. Using this stricter criterion (see the detailed description of diagnostic classifications below) may have masked the result obtained. For example, 61 participants had hyperreflexia without concomitant tremors, which considers these patients "without SS". Furthermore, the 13 participants classified as "without SS" had inducible clonus without simultaneous agitation, diaphoresis, or hyperthermia [75].

These studies pointing to correlations between SS and polymorphisms in CYPs, or 5-HT2A receptors, suggest that more than one polymorphism is related to a higher risk of developing SS symptoms [76].

Diagnosis

**Figure 4:**
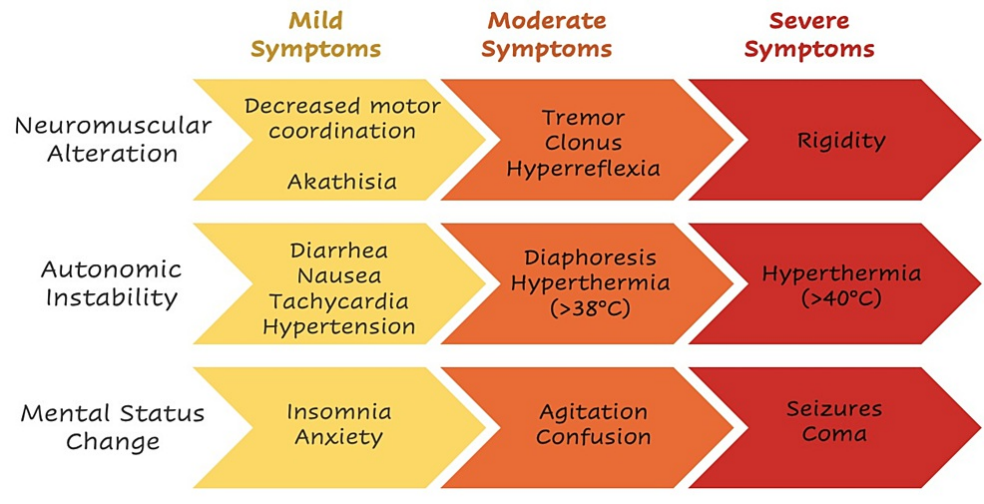
Signs and symptoms of SS within the severity spectrum Starting with mild symptoms (yellow), going through moderate symptoms (orange), and reaching severe symptoms (red). Adapted from [37] and [81].

As shown in Figure 4, the symptoms can range from mild to severe, with potentially fatal forms. Mild symptoms are flu-like and easily ignored.

As it is a purely clinical diagnosis syndrome, it is essential to have a diagnostic algorithm. Currently, three diagnostic classification systems are available: I) the Sternbach Criteria, II) the Radomski Criteria, and III). the Hunter Serotonin Toxicity Criteria. Each system tries to list the characteristic SS symptoms [5].

Sternbach proposed the first diagnostic criteria in 1991. These were based on 3 or more most described symptoms in 38 SS cases [82] presented in Table 3. However, these symptoms (e.g., confusion, agitation, and fever) can be observed in several other clinical conditions, which may indicate a false positive for SS [3,81].

**Table 3:** Sternbach criteria. Adapted from [82].

Sternbach Criteria	
Signs and Symptoms (3 or more)	Confusion or hypomania
	Agitation
	Myoclonus
	Hyperreflexia
	Diaphoresis
	Tremor
	Shivering
	Fever
	Incoordination
	Diarrhea
Inclusion Criteria	Coincidence with the addition or increased dose of a known serotonergic agent within an established therapeutic regimen
	Exclusion of other etiology (e.g., infection, substance abuse, etc.)
	A neuroleptic drug was not started, or at an increased dose, before the onset of the signs and symptoms

The second classification system was developed by Radomski et al. (2000), through the review of 24 additional cases to those analyzed by Sternbach, from 1991 to 1995. In this classification, the cases were divided into I) a moderate state of problems related to 5-HT, II) SS, and III) a toxic state [83]. However, this classification tool, like Sternbach's, also generates false positive results because it excludes symptoms specific to SS.

The Hunter Serotonin Toxicity Criteria are based on data collected from a regional toxicology unit called the Hunter Area Toxicology Service (HATS), which serves the entire Hunter Division, Australia population. All patients admitted to HATS from January 1987 to November 2002 and diagnosed with an overdose of only one serotonergic drug were included in the study (n=473 cases) [3]. As exemplified in Figure 5, based on the statistically significant symptoms presented by these patients, a diagnostic algorithm was produced as a flowchart. This algorithm has improved sensitivity (69%) and specificity (97%) compared to the Sternbach criteria [3,5].

Figure 5: Hunter serotonin toxicity criteria

Adapted from [3], [37] and [39].

However, despite the apparent superiority of the Hunter criteria, the gold standard for SS diagnosis, there is no official consensus on which criterion should be used since the algorithm was developed from cases of overdose by a serotonergic agent, and this may make it less appropriate for diagnosing cases in which there is no overdose [5,84]. The work of Culbertson et al. (2018) suggests that the lower specificity of the Sternbach diagnostic criterion may be adequate for detecting mild to moderate SS cases and that the Hunter criterion is more appropriate for severe SS manifestations [57].

Differential diagnosis 

Clinical diagnosis of SS can be challenging due to comparison with other syndromes (Table 4) and infections (such as meningitis and encephalitis [1]), that have very similar symptoms, which makes it difficult to distinguish between them. Moreover, changes in mental status can present symptoms that mirror those observed in alcohol and drug withdrawal [4,81].

**Table 4:** Comparison between SS and other syndromes Adapted from [1] and [37].

Syndrome	Causative Agent	Vital Signs	Mental Status	Other clinical symptoms
Serotonergic Syndrome	Serotonergic Drugs	Hyperthermia (>40°C), tachycardia, hypertension, and tachypnea	Delirium, agitation, and coma	Neuromuscular hyperactivity (tremor, myoclonus, hyperreflexia, clonus), diaphoresis, and hyperactive bowel sounds
Neuroleptic Malignant Syndrome	Dopamine antagonists and the absence of dopamine	Hyperthermia (>41.1°C), tachycardia, hypertension, and tachypnea	Delirium, agitation	Neuromuscular hypoactivity (rigidity and bradykinesia), hypoactive bowel sounds
Anticholinergic Syndrome	Anticholinergic agents	Hyperthermia (<38.8°C), tachycardia, hypertension (mild), and tachypnea	Hypervigilance, agitation, hallucination, delirium with grumbling, and coma	Normal muscle tone and reflexes, urinary retention, hypoactive bowel sounds, dry skin and mucous membranes
Malignant Hyperthermia	Inhalational anesthetics and depolarizing neuromuscular blockers (succinylcholine)	Hyperthermia (may be greater than 43°C), tachycardia, hypertension, and tachypnea	Agitation	Rigor-mortis-like rigidity, hyporeflexia, skin with signs of flushing and cyanosis, hypoactive bowel sounds

Table 4, which compares the syndromes and clinical conditions that may generate more uncertainties at the time of diagnosis, demonstrates that hyperreflexia and clonus are unique SS symptoms, as advocated by Hunter's criterion, a possible differential. However, not all patients will present these symptoms, which may be related to 5-HT1A receptor agonism [5,37]. About 50% of patients have symptoms related to augmented neuromuscular activity, such as tremors, hyperreflexia, hypertonia, myoclonus, and ataxia [85]. Symptoms related to autonomic instability are present in 40% of patients and include tachycardia, tachypnea, diarrhea, diaphoresis, and increased body temperature [3]. Ignoring a possible case of SS because the symptoms do not meet the Hunter criteria can be a harmful and potentially less safe decision for the patient [5,84].

Serotonin syndrome versus neuroleptic malignant syndrome

Neuroleptic malignant syndrome (NMS) is a significant confounding factor for SS diagnosis [1,5,86]. This syndrome is caused by an adverse reaction to excessive DA D2 receptor blockade or genetically reduced function of this receptor [87]. According to the Diagnostic and Statistical Manual of Mental Disorders (DSM-5), NMS incidence in patients using antipsychotics varies between 0.07% and 1.4% [87]. Despite being a rare syndrome, NMS is better known and present in physicians' memory than SS, which could be because NMS is described in DSM-5, and SS, unfortunately, is not.

A vital factor in differentiating the syndromes is the class of drug that initiated the symptoms. While in SS, they would be serotonergic agents, in NMS, they are antipsychotics [86,88]. However, since second-generation antipsychotics (atypical) act by blocking DA D2 receptors and serotonergic 5-HT2A receptors (and some of them also can act as 5-HT1A agonists [89]), this differentiation factor may be outdated.

Symptoms are also used to guide the differential diagnosis of the two syndromes; for example: in NMS, patients may have stiffness, rhabdomyolysis (characterized by muscle fiber destruction), and increased serum creatine phosphokinase (CPK) levels, which should not occur in SS [90]. However, some SS case reports show that patients present rigidity, sometimes associated with hyperreflexia [91-94] and increased serum CPK levels [92,95,96]. In the meta-analysis carried out by Werneke et al. (2016), with all cases of SS published in the PubMed databases and Thomson Reuter's Web of Science from 2004 to 2014, they observed that in the 299 cases of SS included in the study, 45.4% (136 cases) had stiffness, and 14% had rhabdomyolysis (42 cases) [5].

Another critical point for differentiating the syndromes is the speed with which the symptoms appear after introducing the new drug to the therapy. Many authors support that SS symptoms appear in less than 24 hours and that in NMS, symptoms present between 24 and 72 hours [1,86]. However, some studies have demonstrated that this no longer seems accurate since many patients may present new SS-related symptoms after a week of the new therapy [5,91,95,97].

It is known that MAOIs have a very potent effect due to their mechanism of action that inhibits the main pathway of 5-HT metabolism. Thus, some authors argue that the interaction between an MAOI and an SSRI is more likely to cause more severe and even fatal cases of SS [2,97]. Fox et al. (2007) demonstrated that administering tranylcypromine and 5-HTP (or clorgyline and 5-HTP) was necessary for SS symptoms to appear in mice with normal SERT functioning. In cases where only MAOIs were administered, only SERT knockout mice presented SS symptoms [98]. Fox et al. (2007) demonstrated that administering tranylcypromine and 5-HTP (or clorgyline and 5-HTP) was necessary for SS symptoms to appear in mice with normal SERT functioning [98]. It is important to note that central SS studies, still used as a reference today, were carried out when MAOIs were more frequently prescribed [1,39,44,82].

Therefore, this delay in the appearance of SS symptoms may be due to the change in the pharmacological profile of drugs currently prescribed. This result may modify that well-established temporal parameter of the fastest initiation of SS symptoms. In addition, Werneke et al. (2016) demonstrated that the dose and speed of drug distribution might cause a more rapid or slower syndrome onset [5].

Given all these similarities and difficulties in differentiating these two syndromes, some authors consider them part of the same pathological spectrum [5,99]. Interestingly, some case reports reported a different presentation of NMS (atypical NMS), which does not necessarily present rigidity or hyperthermia as primary symptoms [100,101]. These cases always occur in the presence of atypical antipsychotics. One of the most widely accepted hypotheses about this "atypical" NMS pathophysiology is that, due to the action of these drugs on the serotonergic system, in addition to the lower degree of dopaminergic receptors antagonism, the motor symptoms could be attenuated. In addition, adrenergic and muscarinic receptor activity disruption can lead to autonomic dysfunction [100-102]. Therefore, SS and NMS may be part of the same pathological spectrum since the atypical NMS seems to occur due to an augmented serotonergic action. Indeed, as previously mentioned, some SS cases present NMS symptoms.

Treatment and prevention

The first and foremost treatment of the syndrome is removing the causative serotonergic agent. The symptoms of SS disappear spontaneously in 70% of the cases, with only the withdrawal of the drug(s) involved. The level of additional support will be given depending on the severity of the patient's symptoms; in general, 40% of patients end up needing intensive care, with 25% requiring drastic measures such as orotracheal intubation [44,84].

In addition to withdrawal from serotonergic agents, some mild cases also require benzodiazepines to improve akathisia and anxiety. Benzodiazepines can also be used in cases where the patient has myoclonus, muscle rigidity, and seizures. Beta-blockers (e.g., propranolol and pindolol) also block 5-HT1A receptors, which would be beneficial in moderate cases with hypertension [36,84]. However, animals treated with propranolol did not have an adequate response to SS [103]. 

In the most severe SS cases, often considered a medical emergency, some authors advocate administering cyproheptadine (a non-selective 5-HT1A and 5-HT2A receptor antagonist) if benzodiazepines fail to control symptoms [1,37,104]. This treatment neutralizes excess 5-HT in the synaptic cleft [105]. However, some researchers point out that there is no significant difference in the outcome of treatment with cyproheptadine compared to supportive therapy without this antagonist [36,38,95].

In patients with elevated body temperature caused by SS, cooling measures and reducing muscular activity is an effective treatment strategy [1,104]. This is because the temperature increase is not regulated by the hypothalamic thermostat, but rather by hyperexcitability and direct serotoninergic effects on the muscle [106]. As a result, antipyretics are not recommended for this condition [1,104,106]. However, cyproheptadine may be helpful in these cases as it targets the 5-HT2A receptors, which have been implicated in hyperthermia in animal models [103].

While most cases resolve spontaneously, there are commercially available extended-release formulations releasing the drug associated with SS slowly over time, which may change this estimate. In addition, it is always necessary to consider these drugs' half-life and whether their metabolites have pharmacological effects [37].

The main form of SS prevention is knowledge of its possible causative agents and symptoms by the entire care team, physicians, and patients [1,4,52]. In 1999, Mackay, Dunn, and Mann reported that 85% of physicians across England who prescribed nefazodone within one year (1996-1997) were unfamiliar with SS as a clinical diagnosis [6]. In 2009, a lack of medical awareness was also observed, where seven SS cases were initially misdiagnosed (due to the symptoms presented) in the emergency department of an Israeli hospital. Later, the correct diagnosis was made when the patients showed more classic and severe SS symptoms, including clonus and hyperreflexia [4]. This case demonstrates how unawareness can be harmful because if professionals are not attentive to SS and do not relate the symptoms with the syndrome, the patient will not be treated appropriately. Sometimes, mild and moderate cases will naturally evolve, increasing the syndrome's severity.

## Conclusions

SS is commonly associated with patients receiving treatment for psychiatric disorders. However, since antidepressants are also used to treat other medical conditions, all physicians (not only psychiatrists and neurologists) who prescribe drugs that can induce SS should be educated about this syndrome. Moreover, providing education to patients who are starting antidepressant treatment about the potential adverse reactions and the risk of developing SS is crucial.

The appearance of the syndrome's symptoms in some patients, despite others with very similar conditions, leads us to consider that polymorphisms are the risk factors favoring the increase of 5-HT available in the synaptic cleft. Future studies investigating polymorphisms in critical molecules for the kinetics and/or targets (CYPs, 5-HT receptors, SERT, and MAO) of SS-inducing drugs will yield a greater understanding of the syndrome.
